# Physical Self-Concept, Gender, and Physical Condition of Bizkaia University Students

**DOI:** 10.3390/ijerph17145152

**Published:** 2020-07-17

**Authors:** Iker Sáez, Josu Solabarrieta, Isabel Rubio

**Affiliations:** 1 Faculty of Psychology and Education, Department of Physical Activity and Sport Science, University of Deusto, 48007 Bilbao, Spain; irubio@deusto.es; 2 Faculty of Psychology and Education, Department of Educational Innovation and Organization, University of Deusto, 48007 Bilbao, Spain; josu.solabarrieta@deusto.es

**Keywords:** physical self-concept, physical condition, gender, university students, physical activity, sedentary lifestyle

## Abstract

(1) Background: Despite the benefits of physical activity being well documented in university students, some do not follow the international recommendations. This period of life is a vital stage in adhering to healthy habits in adult life. The objective of the study was to analyze university students’ scores of their physical self-concept and its relationship with gender, physical condition, and level of self-perceived competence. (2) Methods: The sample comprised of 1289 Bizkaia University students (42.12% men and 57.87% women), between 18 and 46.5 years old (M = 20.4; SD = 2.2 years). Physical self-concept, physical condition, number of hours per week of physical activity, and perceived fitness level were analyzed. (3) Results: We found significant differences between women and men in their physical self-concept, but it seemed to be mainly an indirect effect through the mediation of hours of exercise and physical condition. (4) Conclusions: To understand the variance of the level of physical self-concept between genders in university students, the effect of certain variables (physical condition, number of hours per week, and perceived fitness level) must be considered, as well as the mediating role of some of these variables.

## 1. Introduction

The benefits of having a physically active lifestyle during different stages of life are well documented [1]. According to the Physical Activity Guidelines Advisory Committee [2], these include improving cardiorespiratory and muscular health, bone and cardio-metabolic health, and positive effects on cognitive and emotional or social condition. Current evidence suggests that many of these health benefits extend into adulthood [3]. To achieve these benefits, the international recommendations from WHO guidelines [4] require adolescents to engage in 60 min or more of moderate to vigorous physical activity every day, and at least 150 min for adults. Additionally, it also corroborates the notion that a sedentary lifestyle is one of the main health problems among the world population in the 21st century [5]. Recognizing the importance and urgency of the insufficient global levels of physical activity, WHO Member States approved a Global Action Plan on Physical Activity. At the 2018 World Health Assembly, WHO Member States agreed to a new goal of 15% relative reduction in insufficient physical activity among adolescents by 2030 [4].

The negative consequences of increased rates of pathologies, such as obesity, cardiovascular diseases, osteoporosis, type II diabetes, as well as different types of cancer (which implies a sedentary lifestyle), add to a special interest in promoting and maintaining habits adhering to physical activity. Furthermore, the university stage is considered “fragile” because students are prone to losing their previous habits of physical activity [6]. The scientific literature confirms that during the university stage, students experience reduced levels of physical activity during their leisure time—below recommended levels—and increased use of inactive means of transportation, increased time using technological devices [7,8,9], behaviors associated with a lack of time, new schedules, and university commitments [10].

In analyzing the difference in the amount of physical activity between university students based on gender, various studies have confirmed that women are less active than men. Some authors agree on the existence of differences in engaging in physical activity and sports based on gender, establishing that men are 20% more active compared with women [11,12,13]. Research conducted in Japan also confirmed that 61.3% of women, compared with 46.7% of men, do not engage in any type of physical activity [14].

In the university stage, these changes come from the need to face situations that they have not previously encountered. During this time, family habits are partially abandoned due to changes in how students’ tasks and lives are organized. The many fast food options, lack of time, stress, and inexperience in planning an independent life are among other factors [15,16]. The university stage, therefore, becomes a particularly critical moment for people because they will acquire new habits and reinforce others (positive or negative), which will have a significant impact on their future lives. Looking at the positive side of this change, we should take advantage of this stage as it provides us with ideal conditions for establishing healthy lifestyles [17,18]. Therefore, studying and promoting physically healthy habits at the university stage is essential, not only as an investment in current improvement but also to adhere to habits that will be maintained in adult life [9].

Research on the levels of physical activity and physical self-concept is important because of the lasting impact that both constructs generate in subsequent healthcare trajectories, and, more specifically, in studying the importance that the university stage has on adhering to life habits in adulthood [19]. We can define self-concept as the mental image of what people think of themselves, which is made up of different domains [20]. It is related to psychological functioning and well-being throughout life. Therefore, it is important to understand how and when the self-concept’s different domains begin to stabilize, and whether they remain stable throughout childhood, adolescence, and early adulthood [21].

The physical self-concept is one of the main domains of the general self-concept [22]; it is not only related to fitness but is also transferred to various fields such as academic and social aspects [23]. The physical self-concept is understood as a set of physical self-perceptions that would be hierarchically structured in four dimensions: physical ability, physical condition, physical attractiveness, and strength [24]. A better perception of one’s physical self-concept can work as a facilitator of physical activity and as a result of physical activity [25]. The objective of this study is to analyze university students’ physical self-concept scores by using the Physical Self-concept Questionnaire (PSQ) [26]. This will allow us to study the explanatory capacity of gender and other variables, and to examine the mediating role of physical condition on the effect of physical self-concept.

## 2. Materials and Methods

### 2.1. Subjects and Design

A study was conducted using a sample of 1289 university students enrolled at Bizkaia in different university programs, between the ages of 18 and 46.5 (M = 20.4; SD = 2.2 years). Respondents voluntarily participated after receiving a detailed explanation of the objectives and nature of the study. Written informed consent was provided. Seventeen participants who did not correctly meet the inclusion criteria (i.e., they did not complete questionnaires or submit the informed consent forms) were excluded from the analysis. Therefore, the final sample comprised of 1272 subjects (543 men and 729 women). At the time of completing the questionnaire, the students were enrolled at the University of Deusto, and the University of the Basque Country.

### 2.2. Instruments

To measure physical self-concept, the PSQ [26] was administered, as it is a model created in Spanish rather than a translation. However, it is supported by Fox’s physical self-concept model [27]. This questionnaire comprises 36 items and six scales: physical ability, physical condition, physical attractiveness, strength, general physical self-concept, and general self-concept. Only the items referring to the general physical self-concept and physical condition scales were used for this study. Of the 12 items (six per scale) presented, with five response options on a Likert scale with scores from 1 to 5 (from false to true), some are written directly (“I have a lot of physical strength”) while others are indirect (“I feel unhappy with myself”). The reliability coefficient (Cronbach’s alpha) of the physical self-concept subscale was α = 0.860 and α = 0.880 for physical condition.

We measured the type and volume of physical activity and its perceived level by using direct questions: “In total, how many hours of each type of exercise did you participate in, or practice per week? (Includes games, training sessions, matches..).”; “Indicate the level (skill, ability..). that you think you had or have in this type of exercise from 0 to 10.”

### 2.3. Procedure

First, approval for the study was requested from the University of Deusto Ethics Committee, which was granted with the code “ETK-24/17-18”. Once the questionnaire was created and ethical appropriateness was confirmed, collaboration was requested from both the University of Deusto and the University of the Basque Country. After obtaining permission, spaces to administer the questionnaire were requested.

Data were collected during student breaks between classes in different university campuses. To collect the data and to ensure that the questionnaires were correctly administered and completed, the researcher in charge was present to resolve any issues that may have arisen. At that time, prior to administering the questionnaire, all participants were instructed that participation was voluntary and that the data collected would remain confidential. The participants did not receive any type of incentive. The data were collected between February and March 2018.

### 2.4. Statistical Analysis

Descriptive analyses, t-tests, and correlations were calculated using IBM SPSS software (v.26). The effect size, reflected in the mean differences, was estimated using Cohen’s d. A value of 0.2 was considered “small”, 0.5 “medium” and 0.8 “large” [28]. The level of significance used was 0.05.

IBM AMOS software (v.26) was used to analyze the relationships between the constructs involved using a path analysis.

## 3. Results

A total of 321 (25.9%) subjects said that they do not exercise at all, while 916 subjects (74.5%) exercised in some capacity. The average weekly amount of time dedicated to physical activity was 5.35 h (SD = 4.82) in the entire sample and 7.22 h (SD = 4.22) among people who did exercise. Physical condition, physical self-concept and perceived level of exercise have a negative skewness, caused by the accumulation of cases at the highest values. On the other hand, in the weekly hours the asymmetry is positive, due to the fact that people who do less physical activity accumulate in a narrow strip of the lower zone of the distribution, near the value 0. These are important values of skewness, but the statistics used are sufficiently robust in this respect (Table 1).

The group of men obtained a significantly higher mean for physical condition than the group of women with a large effect size (d = −0.79). The difference in physical self-concept was also statistically significant, but with a medium effect size (d = −0.44). Weekly average hours of exercise were 3 h higher in the group of men. However, there were no statistically significant differences in the mean for the perceived level between the two groups (Table 2).

A vertical reading of the means of physical self-concept of active and sedentary men and women does not show a great difference between men and women in the effect of activity on physical self-concept (Table 2). This effect may be due to the fact that other dimensions (physical attractiveness, strength, etc.) in turn have a greater effect on the relationship between gender and physical self-concept. We calculated the correlations between the number of hours per week of exercise, physical condition, physical self-concept, and perceived level of exercise (Table 3).

Given the existence of a relevant subgroup of people whose number of hours is zero, causing strong asymmetry in this variable’s distribution, we calculated these correlations twice. First, we used the entire sample and included people who do not exercise (represented above the diagonal), and then calculated again with only those who exercise (represented below the diagonal).

The differences between the coefficients on both sides of the diagonal are small. Therefore, the correlations found are stable, both in the entire sample and in the group of people who exercise. The weekly hours of exercise have a relevant correlation with physical condition but to a lesser extent with physical self-concept and even less so with the perceived level of exercise. When we balanced the effect of the gender variable, we did not find important differences in the correlations of the women’s and men’s groups. The correlation between weekly hours of exercise and physical condition is weaker when the two sexes are considered separately. This lesser correlation may indicate that the gender variable is simultaneously conditioning both hours of exercise and physical condition.

Physical condition is related to physical self-concept much more strongly than to perceived level of exercise, both in women and men.

The relationship between perceived level of exercise and physical self-concept is limited similarly in women and men (Table 4 and Table 5).

We conducted a path analysis gathering all the variables studied. This allows us to differentiate the direct and indirect effects.

**Figure 1:**
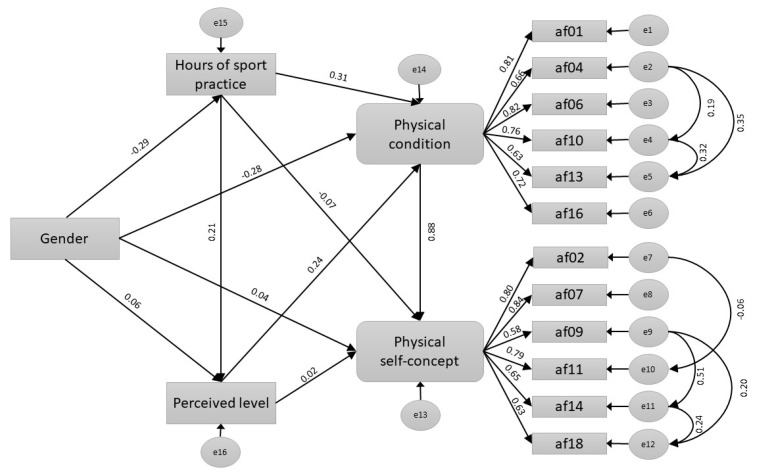
Path analysis showing the physical self-concept variance according to gender, hours of exercise, physical condition, and perceived level.

The resulting model (Figure 1) has adequate goodness of fit indices (RMSEA = 0.073).

We did not find a statistically significant direct effect of sex on perceived level (*p* = 0.152) or on physical self-concept (*p* = 0.706), nor on perceived level on physical self-concept (*p* = 0.726). However, when we look at the value of the total effect of sex on physical self-concept, it is statistically significant (−0.280), so it is possible that there are important indirect effects (−0.290). The total effect of sex on perceived level remains irrelevant (0.004), therefore, we ruled out indirect effects of sex on perceived level (Figure 1).

The indirect effect of sex on self-concept seems to occur through three paths. All three effects are statistically significant (*p* = 0.001). These include the indirect effects that occur through physical condition (−0.443); exercise and physical condition (−0.091); and through exercise, perceived level, and physical condition (−0.018) (Figure 1).

## 4. Discussion

The benefits that physical exercise offers have always been an extremely attractive topic in the field of health and quality of life, especially in university-age people, which is a crucial time period for adhering to definitive habits that will be maintained in adult life [17,18,19,20,21,22,23,24,25,26,27,28,29]. Therefore, physical self-concept is relevant, as people’s motivation to continue being physically active is influenced by their perception of physical self-concept, as it changes because of physical activity [30]. The objective of this study was to analyze university students’ physical self-concept scores using the PSQ [26] to study the explanatory capacity of sex and other variables, and to examine the mediating role of physical condition on the effect of other physical self-concept variables.

The results obtained show us that 25.9% of the sample is inactive; however, there are studies [31,32,33] that provide more concerning data, stating that in European countries, the rate of inactive university students varies between 35% and 89%. On the contrary, research carried out with Spanish university students concluded that a high percentage complied with the recommendations of the International Organizations [6]. A possible explanation for the data obtained in our study is due to the fact that the sample collected to compare with other research studies [34], a high percentage (27.9%) comprise students in Sports Science degree programs with a very low sedentary level, while the level of inactivity in the rest of the population may be closer to the other studies consulted. As in other research [35,36], it is evident that students in health-related careers are more compliant with recommended levels of physical activity.

In relation to the number of hours of exercise and gender, men show more motivation in terms of physical activity compared with women, and various studies confirm this [37,38,39,40,41,42,43]. Men also had higher scores for perception of physical condition. The difference is large (d = −0.79), which is in line with other works [32,44,45,46]. As we can see in other studies, other dimensions of the physical self-concept (physical attractiveness, for example) have a greater effect on women [47,48,49]. However, research carried out with Spanish students in Universities where students share university sports programs, with common calendars, schedules and spaces, men and women show similar levels of physical activity [50,51]. In the case of the participants in this study, these shared programs are not available to students, men perform activities of higher intensity and organized and women of lower intensity and not organized [52].

In this study, we found slightly higher mean values among men in the dimensions measured by the PSQ [26] (d = −0.44). Studies carried out by various authors [53,54,55,56] obtained similar results to those deduced in this work, as their figures were lower for women in perceived physical condition due to the difference in habits between both genders. Students with higher physical self-concept have a better perception of their own health and take care of their eating and resting habits [57]. Especially in the case of women, it is important not to fall into social comparisons or stereotypes in order to achieve adherence to habits that enhance their physical self-concept [44,58].

Additionally, the results of this study show that the direct effect between gender and physical self-concept (0.04) is weak and slightly higher for women, just as observed in previous studies carried out [49,59,60] and contrary to the data provided by other published studies [30,61,62,63]. In contrast, the indirect effect is much stronger and favors the men’s group, which is in line with various studies [64,65,66,67,68,69]. The path analysis shows that a path between gender and physical self-concept using the number of hours of exercise and the physical condition dimension has a very strong effect. The results of our study’s multivariate analysis show a statistically significant effect of interaction between gender, the number of hours of exercise, physical condition, and physical self-concept.

## 5. Conclusions

To conclude, a low percentage of the university students surveyed lead sedentary lifestyles. However, there are differences between women and men in relation to the volume/number of hours of weekly physical activity, which is higher among men. With regard to the relationship between gender and physical condition, there are differences between men and women, likely due to the greater number of hours of exercise.

The difference between women and men for the perceived exercise level variable is not noteworthy. This may be because of the fact that women who exercise in the university stage have overcome barriers (stereotypes, social pressure, etc.) that make their perceived level high. The results obtained allow us to observe that there are no differences in the physical self-concept between university students based on gender. Moreover, the relationship between both variables is conditioned by multiple indirect effects.

In light of the results, it seems necessary to strengthen programs that promote physical activity among university students. Due to the changes in their lifestyle and the emergence of new responsibilities, they may abandon practices they followed as adolescents. This should be emphasized even more strongly among female students.

## Figures and Tables

**Table 1:** Sample size, mean, and deviation of physical condition, physical self-concept, weekly hours of exercise, and perceived level of exercise.

	N	Mean	SD	Skewness	Cronbach’s Alpha
Physical condition	1272	3.35	0.97	−0.29	0.880
Physical self-concept	1271	3.74	0.80	−0.59	0.860
Weekly hours of exercise	1237	5.35	4.82	0.84	-
Perceived level of exercise	913	7.67	1.33	−0.99	-

**Table 2:** Difference of means, standard deviation, Cohen’s d, t-test of the sample in physical condition, physical self-concept, weekly hours and perceived level, and differentiating between active and sedentary people.

	Female		Male					
	Mean	SD	Mean	SD	Cohen’s d	*t-*Test	df	*p*-Value
Physical condition (entire sample)	3.02	0.92	3.78	0.85	−0.79	15.11	1270	0.000
- (active)	3.19	0.86	3.86	0.81	−0.74	12.20	902	0.000
- (sedentary)	2.65	0.91	3.46	0.93	−0.82	7.25	315	0.000
Physical self-concept (entire sample)	3.59	0.78	3.94	0.78	−0.44	7.84	1269	0.000
- (active)	3.68	0.76	4.00	0.73	−0.42	6.49	901	0.000
- (sedentary)	3.37	0.74	3.65	0.92	−0.35	2.89	315	0.004
Weekly hours of exercise	4.13	4.18	6.99	5.13	−0.50	10.78	1235	0.000
Perceived level of exercise	7.68	1.28	7.66	1.39	0.09	–0.26	911	0.798

**Table 3:** Correlations between weekly hours, physical condition, perceived level, and physical self-concept (entire sample).

	Weekly Hours of Exercise	Physical Condition	Perceived Level of Exercise	Physical Self-Concept
Weekly hours of exercise	-	0.41 **	0.17 **	0.31 **
Physical condition	0.37 **	-	0.21 **	0.61 **
Perceived level of exercise	0.18 **	0.21 **	-	0.25 **
Physical self-concept	0.27 **	0.61 **	0.25 **	-

Notes: ** correlation is significant at the 0.01 level (two-tailed).

**Table 4:** Correlations between weekly hours, physical condition, perceived level, and physical self-concept (females).

Women	Weekly Hours of Exercise	Physical Condition	Perceived Level of Exercise	Physical Self-Concept
Weekly hours of exercise	-	0.36 **	0.19 **	0.26 **
Physical condition	0.30 **	-	0.25 **	0.57 **
Perceived level of exercise	0.19 **	0.26 **	-	0.27 **
Physical self-concept	0.22 **	0.55 **	0.27 **	-

Notes: ** correlation is significant at the 0.01 level (two-tailed).

**Table 5:** Correlations between weekly hours, physical condition, perceived level, and physical self-concept (males).

Men	Weekly Hours of Exercise	Physical Condition	Perceived Level of Exercise	Physical Self-Concept
Weekly hours of exercise	-	0.31 **	0.16 **	0.26 **
Physical condition	0.29 **	-	0.21 **	0.62 **
Perceived level of exercise	0.20 **	0.19**	-	0.24 **
Physical self-concept	0.23 **	0.64 **	0.25 **	-

Notes: ** correlation is significant at the 0.01 level (two-tailed).
